# BMP and activin membrane-bound inhibitor regulate connective tissue growth factor controlling mesothelioma cell proliferation

**DOI:** 10.1186/s12885-022-10080-x

**Published:** 2022-09-15

**Authors:** Nguyen Truong Duc Hoang, Ghmkin Hassan, Tomoya Suehiro, Yuichi Mine, Tohru Matsuki, Makiko Fujii

**Affiliations:** 1 Department of Genomic Oncology and Oral Medicine, Graduate School of Biomedical and Health Science, Hiroshima University, 1-2-3 Kasumi, Minami-ku Hiroshima-city, 834-8553 Japan; 2 Department of Medical System Engineering, Division of Oral Health Sciences, Graduate School of Biomedical and Health Sciences, Hiroshima University, Hiroshima-city, Japan; 3 Department of Cellular Pathology, Institute for Developmental Research, Aichi Developmental Disability Center, Kasugai-city, Aichi Japan

**Keywords:** CTGF, BAMBI, Malignant mesothelioma, Proliferation; Cell cycle

## Abstract

**Background:**

Malignant mesothelioma (MM) is an aggressive mesothelial cell cancer type linked mainly to asbestos inhalation. MM characterizes by rapid progression and resistance to standard therapeutic modalities such as surgery, chemotherapy, and radiotherapy. Our previous studies have suggested that tumor cell-derived connective tissue growth factor (CTGF) regulates the proliferation of MM cells as well as the tumor growth in mouse xenograft models.

**Methods:**

In this study, we knock downed the bone morphogenetic protein and activin membrane-bound inhibitor (BAMBI) and CTGF in MM cells and investigated the relationship between both and their impact on the cell cycle and cell proliferation.

**Results:**

The knockdown of CTGF or BAMBI reduced MM cell proliferation. In contrast to CTGF knockdown which decreased BAMBI, knockdown of BAMBI increased CTGF levels. Knockdown of either BAMBI or CTGF reduced expression of the cell cycle regulators; cyclin D3, cyclin-dependent kinase (CDK)2, and CDK4. Further, in silico analysis revealed that higher BAMBI expression was associated with shorter overall survival rates among MM patients.

**Conclusions:**

Our findings suggest that BAMBI is regulated by CTGF promoting mesothelioma growth by driving cell cycle progression. Therefore, the crosstalk between BAMBI and CTGF may be an effective therapeutic target for MM treatment.

**Supplementary Information:**

The online version contains supplementary material available at 10.1186/s12885-022-10080-x.

## Background

Mesothelioma is a rare malignant tumor originating from the mesothelial surface of the pleura or, more rarely, at other locations such as the peritoneum [1]. Inhalation of asbestos dust is the leading cause of malignant mesothelioma (MM), accounting for 80%–90% of all diagnosed pleural mesothelioma cases [1]. Asbestosis was once widely used in building construction but began to be phased out in many industrialized countries during the 1970s and 1980s. However, the latent period from exposure to development of MM is typically 30 to 40 years, so prevalence and death rates remain substantial. Further, prevalence and death rates have continued to increase in developing countries where asbestos remains in use [2]. Most patients are diagnosed in the late stage, by which the time median survival period is only 12 to 20 months [3]. The current first-line chemotherapy for mesothelioma is a combination of cisplatin and pemetrexed, but this regimen is only a modest benefit for overall survival [4]. Several drugs targeting the signaling pathways underlying MM are currently under evaluation, but clinical trials have not demonstrated significant benefits [5]. Therefore, more detailed analysis of the signaling mechanisms underlying MM progression are needed to identify novel molecular targets for improved therapy.

Recent studies have identified cell-derived connective tissue growth factor (CTGF) as a novel therapeutic target for the treatment of multiple human diseases, including various types of cancers [6, 7]. CTGF, a member of the CCN cysteine-rich family, is a 36–38 kDa multifunctional secretory protein involved in numerous cellular processes relevant to cancer including angiogenesis, cell proliferation, apoptosis, fibrosis, inflammation, epithelial-to-mesenchymal transition (EMT), and tissue invasion [8–10].

Our previous study suggested that CTGF expression is regulated by the crosstalk between Hippo and transforming growth factor beta (TGF-β) signaling pathways in MM cells, as both blockade of TGF-β signaling and suppression of CTGF protein expression reduced MM growth [11, 12]. There are other reports which also showed the association between CTGF and poor prognosis of MM [13–15]. Based on these findings, we started to explore the downstream gene target of CTGF which might take responsibilities in regulation of cell proliferation and malignancies in MM cells.

BAMBI was initially described as a pseudoreceptor inhibitor of the TGF-β/bone morphogenic protein (BMP) signaling pathway based on the structural homology with TGF-β family type I receptors and lack of an intracellular serine/threonine kinase module [16]. While BAMBI has been implicated in cancer progression, these tumorigenic functions appear to be highly context-dependent [17]. For instance, elevated BAMBI expression was correlated with poor prognosis in colorectal cancer (CRC), while BAMBI inhibition upregulated TGF-β signaling, resulting in reduced CRC cell viability and motility in vitro and in vivo [18, 19]. Conversely, loss of BAMBI was found to promote lung and bladder tumorigenesis through overactivation of TGF-β1 and acquisition of pro-invasive properties such as induction of EMT and increasing cell mobility and migration [20–22]. CTGF is known to be activated by TGF-β signaling and several studies reported a relationship between BAMBI and CTGF in different disease [23–25]. However, the functions of BAMBI in MM are not yet clarified.

Different cell lines have been isolated from human malignant mesotheliomas and pleural fluids of non-cancerous individuals [26, 27]. In this study, we investigated the molecular targets of CTGF in MM cells to identify additional therapeutic targets. We also explored the relationship between CTGF and BAMBI in MM cells. Our results indicated that inhibition of either CTGF or BAMBI reduced both MM cell proliferation rate and the expression of cell cycle proteins. Further, in silico analysis revealed that elevated BAMBI expression is associated with reduced survival rates in mesothelioma patient, suggesting that BAMBI may be an effective molecular target for MM therapy.

## Materials and methods

### Cell culture

The human mesothelioma cell lines: NCI-H28, NCI-H2052, NCI-H2452, and MSTO-211H, and the simian virus 40 (SV40)-transformed mesothelial cell line MeT-5A were purchased from the American Type Culture Collection (ATCC, Manassas, VA, USA). The other human mesothelioma cell lines used in this study; Y-MESO-8D, Y-MESO-14, and Y-MESO-27, were the gifts from Dr. Y. Sekido, Aichi Cancer Center Research Institute (Aichi, Japan). All mesothelioma cells were maintained in RPMI-1640 medium with L-glutamine and Phenol Red (Fujifilm-Wako, Tokyo, Japan) supplemented with 10% fetal bovine serum (FBS) (EU origin; Biosera) at 37 °C under a 5% CO_2_ atmosphere.

### Small interfering RNA (siRNA) and antibodies

ON-TARGETplus Human CTGF siRNA-SMART pool, ON-TARGETplus Human BAMBI siRNA-SMART pool, and ON-TARGETplus non-targeting Pool siRNA were purchased from Dharmacon (Lafayette, CO, USA) and were used for all transfection experiments. BAMBI siRNA(h) #sc-60243, a pool of 3 target-specific siRNAs (Santa Cruz Biotechnology, Dallas, TX, USA) was only used to confirm the effect of knockdown BAMBI expression on MM cell proliferation. The oligonucleotide sequences of the siRNAs in each pool are shown in (Table S1).

An antibody against BAMBI/NMA (ab203070) was purchased from Abcam (Cambridge, UK), anti-CTGF (sc-14939) and anti-goat IgG (sc-2354) from Santa Cruz Biotechnology (Dallas, TX, USA), and antibodies against cyclin D1 (92G2), cyclin D3 (2936), CDK2 (2546), CDK4 (1279), phospho-Smad2 (Ser465/467), phospho-Smad3 (Ser423/425) (C25A9), Smad2/3, rabbit IgG (7074), and mouse IgG (7076) from Cell Signaling Technology (Danvers, MA, USA). Anti-β-actin (A5441) was obtained from Sigma-Aldrich (St. Louis, MO, USA).

### Cell transfection

The MM cells were seeded in six-well plates with culture medium, incubated overnight under standard culture conditions, and then transfected with the indicated siRNAs using Lipofectamine RNAiMax (Thermo Fisher Scientific, Waltham, MA, USA) according to the manufacturer's protocol. Cells were collected and subjected to qRT-PCR and western blotting. At least three independent replicates were performed for each experiment.

### Real-time quantitative reverse transcription PCR (RT-qPCR) and expression profiling with microarray

Total RNA was isolated using the Nucleospin RNA plus kit (Macherey–Nagel GmbH & Co. KG, Dueren, Germany) according to the manufacturer's instructions. The gene-specific primer sets for RT-qPCR were designed using Primer Express Software Version 3.0 (Thermo Fisher Scientific, Waltham, MA, USA) based on the RefSeqs shown in (Table S2). The first-strand complementary DNA (cDNA) was synthesized using ReverTra Ace qPCR RT Master Mix (Toyobo, Osaka, Japan) and RT-qPCR was conducted with a StepOnePlus Real-time PCR System using PowerUp SYBR Green Master Mix (Thermo Fisher Scientific, Waltham, MA, USA). The gene expression levels were normalized to that for GAPDH expression. The microarray profiling was conducted for Y-MESO-27 cells transfected with shCTGF. Total RNA was extracted from cells and subjected to microarray analysis as described previously [11].

### Western blotting

Cells were washed in ice-cold phosphate-buffered saline (PBS), harvested, and incubated for 20 min., in ice-cold lysis buffer consists of 10 mM HEPES, 200 mM NaCl, 30 mM sodium pyrophosphate, 50 mM NaF, 5 µM ZnCl_2_, and 1.0% Triton X-100, pH 7.5 supplemented with complete mini protease inhibitor cocktail (Roche Diagnostics GmbH, Mannheim, Germany). Cell lysates were centrifuged at 12,000 × g for 20 min., at 4 °C and the supernatant proteins were denatured in SDS sample buffer (Invitrogen, Carlsbad, CA, USA) at 96 °C for 5 min. About 30 μg of total protein per gel lane was separated on 10% Tris-Gly SDS–PAGE gels (Invitrogen, Carlsbad, CA, USA), and subsequently blotted onto Immobilon-P membranes (Millipore, Burlington, MA, USA). Membranes were blocked with 5% non-fat milk in Tris-buffered saline containing 0.1% Tween 20 (TBS-T) for 1 h at room temperature, washed 3 times with TBS-T, and incubated with the indicated primary and secondary antibodies. Protein bands were detected by chemiluminescence using the ChemiDoc XRS system (Bio-Rad, Hercules, CA, USA).

### Immunofluorescence staining

Cells were grown overnight on glass coverslips coated with poly-L-lysine (P2636, Sigma -Aldrich, St. Louis, MO, USA). After washing with PBS, cells were fixed in 4% formaldehyde/PBS for 10 min., and permeabilized with 0.1% Saponin/PBS for 10 min., at room temperature. Subsequently, cells were blocked for 1 h with 5% bovine serum albumin in TBS-T and incubated overnight with anti-BAMBI (Abcam, Cambridge, UK) and anti-N-cadherin (Cell Signaling Technology, Danvers, MA, USA) in blocking buffer at 4 °C. Cells were then washed three times in TBS-T and incubated with the appropriate Alexa-conjugated secondary antibodies (Thermo Fisher Scientific, Waltham, MA, USA) at room temperature. Coverslips were mounted on glass slides with VectaShield mounting medium (H-1000; Vector Laboratories, Burlingame, CA) containing DAPI for nuclear counterstaining. All images were captured using a LSM880 confocal microscope (Zeiss, Jena, Germany).

### Cell viability assay

Cells were seeded in 96-well plates and incubated overnight. The culture medium was then replaced with fresh medium supplemented with siRNAs at the indicated concentration. Cell proliferation was determined by PrestoBlue (Thermo Fisher Scientific, Waltham, MA, USA) according to manufacturer’s instructions at 0, 24, 48, and 72 h after addition of siRNAs. Briefly, at these indicated times, cells were incubated for 2 h in 10% PrestoBlue medium and fluorescence was measured at 590 nm from 546 nm excitation using Varioskan Flash (Thermo Fisher Scientific, Waltham, MA, USA) as an estimate of viable cell number.

### Survival analysis

The influence of BAMBI expression on survival rates of MM patient was analyzed using Gene Expression Profiling Interactive Analysis (GEPIA, http://gepia.cancer-pku.cn/about.html), a web-based tool that can assess RNA sequence expression data from The Cancer Genome Atlas (TCGA) and Genotype Tissue Expression (GTEx) projects [28]. Kaplan–Meier survival analysis was conducted by comparing patients with high and low BAMBI mRNA expression levels using the time from diagnosis to death as the outcome variable. Survival curves were compared by log-rank test, with a *p-value* < 0.05 considered statistically significant.

### Statistical analysis

All results are expressed as mean ± standard deviation (SD) from a minimum of three independent replicates. Statistical differences were determined by one-way ANOVA with post hoc Bonferroni test for three-group comparisons or by two-tailed Student's t-test for two-group comparisons. A *p-value* < 0.05 was considered significant for all tests.

## Results

### CTGF knockdown suppresses MM cell proliferation and BAMBI protein expression

**Fig. 1:**
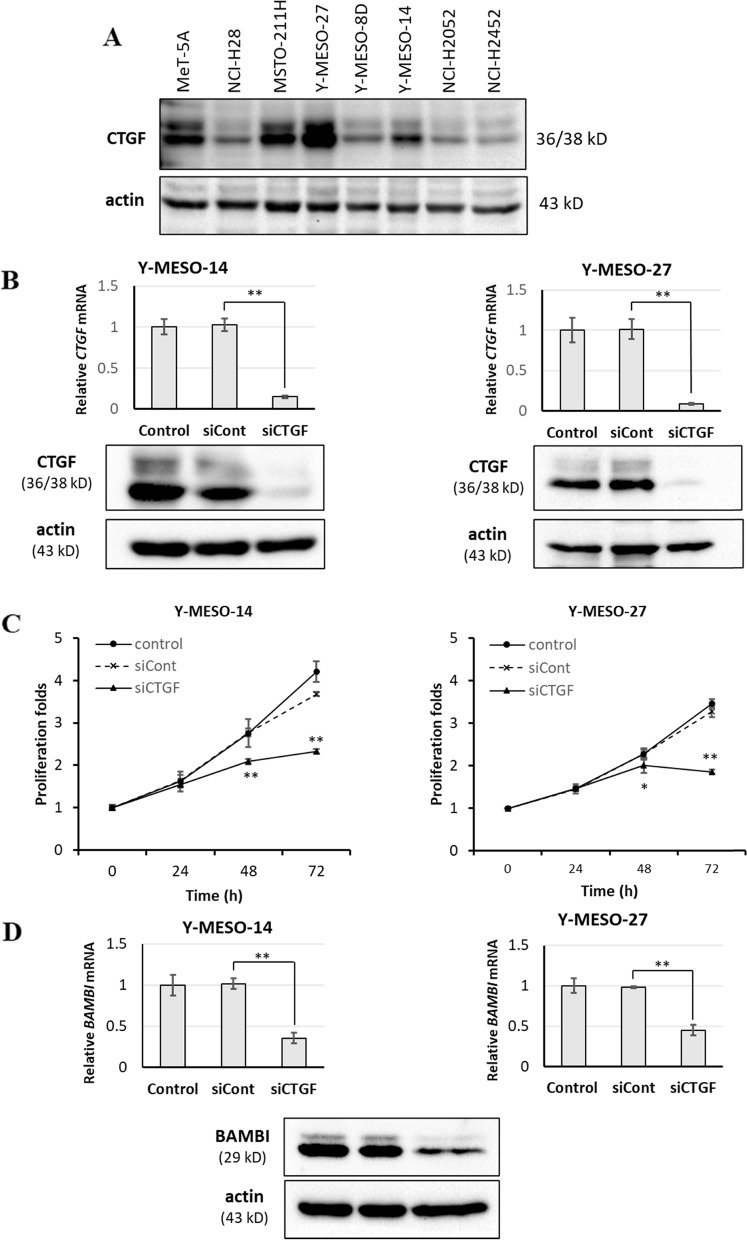
Knockdown of CTGF reduces MM cell proliferation rate and suppress the expression of BAMBI. **A**, Western blots of MM cell lines NCI-H28, MSTO-211H, Y-MESO-8D, Y-MESO-14, Y-MESO-27, NCI-H2052, and NCI-H2452. Antibodies against actin and CTGF were used as the primary antibodies. **B**, Bar graphs of relative expression levels of CTGF in Y-MESO-14 and Y-MESO-27 cells transfected with a CTGF-targeted siRNA (20 nM) and compared with CTGF levels in non-transfected cells and cells transfect with control siRNA. The blots showing protein levels assessed by western blotting in control cells and cells transfected CTGF-targeted siRNA. **C**, Graphs of Proliferation rates as measured by viable cell counting assay. **D**, Graphs of relative mRNA levels and a western blot of BAMBI levels in control cells and cells transfected with CTGF-targeted siRNA. All results are expressed as mean ± SD of three independent experiments (*p < 0.05 vs. control siRNA group by one-way ANOVA with post hoc Bonferroni tests for pair-wise comparisons). Original unprocessed western blots are included in (Supplementary file, original blots)

Our previous studies revealed widely varying CTGF immunostaining intensity in mesothelioma tumor xenografts [11]. Therefore, we assessed CTGF protein expression in multiple mesothelioma cell lines to identify those with high expression for subsequent studies. The MSTO-211H, Y-MESO-14, and Y-MESO-27 cells showed remarkable CTGF protein expression levels compared with other cell line cells (Fig. 1A). MeT-5A cells are commonly used in experiments as a noncancerous mesothelial cell line. MeT-5A cells express CTGF at a certain level. Our previous data revealed that mesothelial cells in normal tissues do not express a high level of CTGF [11]. Since MeT-5A cells are immortalized with the SV40 early region, there might be changes that affects the regulation of CTGF in the genome or intracellular signaling level. This may explain the difference in the expression level of CTGF between normal tissues and MeT-5A cells.

In our previous report, we showed that CTGF not only affects the proliferation of mesothelioma cells and the production of collagen in the microenvironment but also correlates with the malignancy of the disease. CTGF regulates various molecules that vary among different cell types and contexts. Our microarray data also showed that the expression of many genes was changed in CTGF knockdown cells and among these genes, BAMBI was found as a potential TGF-β signaling pathway-related gene, which was downregulated by CTGF knockdown (Table S3). Since our previous report showed that the TGF-β signaling pathway regulated the CTGF expression, we tried to focus on the relationship between CTGF and BAMBI to find the mechanism of MM cell proliferation.

Knockdown of CTGF with CTGF-targeted siRNA showed reduction in CTGF mRNA expression by more than 75% in all examined MM cell lines (Fig. S1, Fig. 1B). Further, western blotting confirmed reduced CTGF protein levels in Y-MESO-14 and Y-MESO-27 cells (Fig. 1B). We also found that knockdown of CTGF in Y-MESO-14 and Y-MESO-27 cells reduced cell proliferation rates measured by cell viability assay compared to cells transfected with a control siRNA (Fig. 1C).

To investigate the mechanism of cell growth regulation by CTGF in mesothelioma cells, we performed cDNA microarray analysis using Y-MESO-27 cells and found that BAMBI gene expression was suppressed by siRNA CTGF (data not shown). RT-qPCR confirmed that CTGF knockdown reduced BAMBI expression in both Y-MESO-14 and Y-MESO-27 cells (Fig. 1D) as well as in four of the five other mesothelioma cell lines subjected to CTGF knockdown (Figure S1). MSTO-211H, Y-MESO-27, and Y- MESO-14 cells exhibited highest levels of reduction of BAMBI expression levels by CTGF knockdown. Western blotting also revealed that CTGF knockdown reduced BAMBI protein expression in Y-MESO-14 and Y-MESO-27 cells (Fig. S1, Fig. 1D). These cells showed previously highest CTGF expression levels and extracellular matrix protein deposition in mouse xenograft models [11]. These findings strongly suggest that BAMBI is a downstream regulatory target of CTGF in MM.

### BAMBI knockdown suppresses MM proliferation and upregulates CTGF

**Fig. 2:**
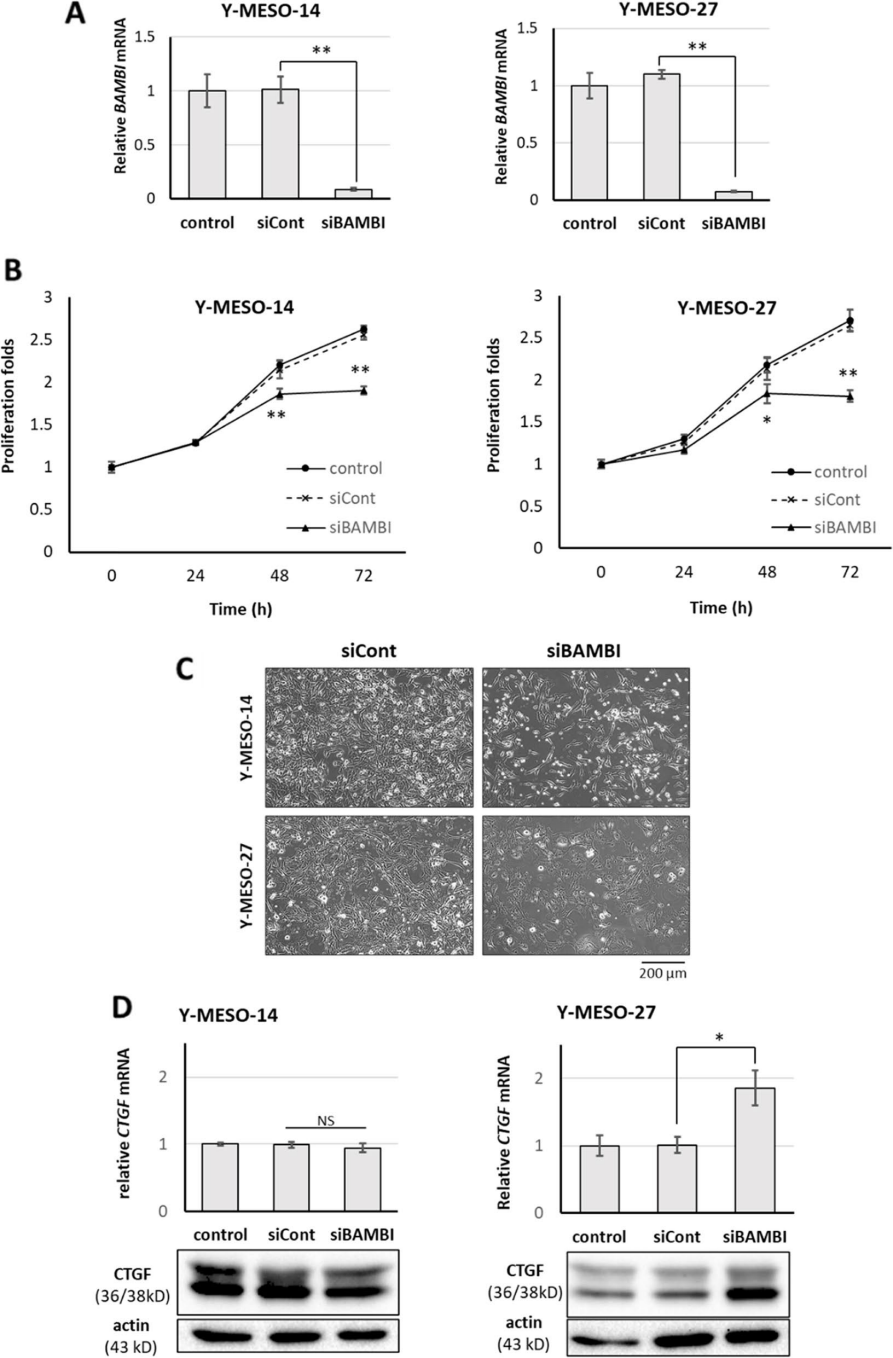
Knockdown of BAMBI suppresses MM cell proliferation and enhances CTGF expression. **A**, Bar graphs of relative expression levels of BAMBI in Y-MESO-14 and Y-MESO-27 cells transfected with a BAMBI siRNA (20 nM) and compared with BAMBI levels in non-transfected cells and cells transfect with control siRNA. **B**, Estimated numbers of viable Y-MESO-14 and Y-MESO-27 cells at 0, 24, 48, and 72 h after BAMBI siRNA transfection. **C**, Representative images of Y-MESO-14 and Y-MESO-27 cell after 72 h of transfection with BAMBI siRNA and control siRNA. Scale bar = 200 μm. **D**, Graphs of relative mRNA levels and western blots of CTGF levels in control cells and cells transfected BAMBI-targeted siRNA as measured by RT-qPCR and western blotting, respectively. All results are expressed as mean ± SD of three independent experiments (*p < 0.05 vs. control siRNA group by one-way ANOVA with post hoc Bonferroni tests for pair-wise comparisons)

To investigate the effect of BAMBI in MM growth and proliferation, we first examined the effect of siRNA-mediated knockdown on the proliferation of Y-MESO-14 and Y-MESO-27 cells. Transfection cells with siRNA significantly downregulated BAMBI expression (Fig. 2A), and similar to CTGF knockdown, concomitantly reduced cell proliferation measured by cell viability assay (Fig. 2B and C). MM cell proliferation was also significantly suppressed by the same procedure with Santa Cruz's BAMBI siRNA pool (Fig. S2). These results suggest that MM growth is controlled by a CTGF/ BAMBI cascade.

TGF-β signaling regulates CTGF expression in MM [11] and BAMBI was initially described as a transmembrane pseudoreceptor for TGF-β/BMP [29], We speculated that BAMBI may exert negative feedback on CTGF expression through disruption of TGF-β signaling. Consistent with this notion, BAMBI knockdown upregulated CTGF mRNA and protein expression in the Y-MESO-27 cell line (Fig. 2D). BAMBI is usually act as TGF-β/Smad inhibitor. Thus, cells were stimulated with TGF-β1 to investigate whether BAMBI acts on TGF-β/Smad signaling in MM cells. The results showed that TGF-β1 activated TGF-β/Smad signaling through phosphorylation of Smad2 and 3, and BAMBI knockdown had no effect on Smad phosphorylation (Fig. S3). Therefore, the data showed that BAMBI expression did not act through the TGF- β/Smad signal pathway in MM cells.

### Knockdown of BAMBI or CTGF downregulates cell cycle regulatory proteins

**Fig. 3:**
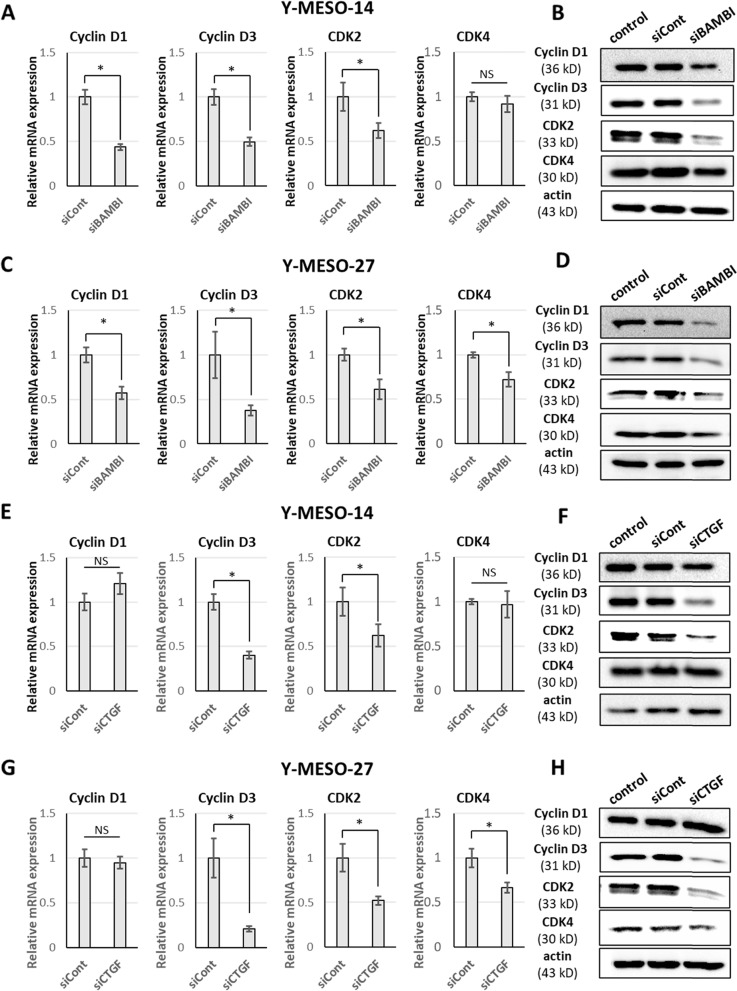
Knockdown of CTGF or BAMBI reduces the expression of cell cycle regulators. **A**, **C**, **E**, **G**, Bar graphs showing relative mRNA levels of cyclin D1, cyclin D3, CDK2, and CDK4 were estimated by RT-qPCR in Y-MESO-14 and Y-MESO-27 cells transfected with BAMBI siRNA (A-D) or CTGF siRNA (E–H) compared with cells transfect control siRNA. Results are expressed as mean ± SD of three independent experiments (*p < 0.05 by two-tailed Student’s t-test). **B**, **D**, **F**, **H**, Cyclin D1, cyclin D3, CDK2, and CDK4 protein expression by mesothelioma cells following BAMBI or CTGF knockdown. Both cyclin D3 and CDK2 expression levels were reduced by either BAMBI or CTGF knockdown

It has been reported that BAMBI modulates the proliferation of human osteosarcoma cells by regulating the expression of cell cycle proteins [30], therefore we examined if BAMBI knockdown influences the expression of cell cycle regulatory molecules in Y-MESO-27 and Y-MESO-14 cells. For cell cycle progression, the D-cyclin family proteins cyclin D1 and cyclin D3 form a complex with cyclin-dependent kinase (CDK)4 and/or CDK6, while CDK2 forms an active complex with cyclin E. Consistent with the effects on proliferation rate, BAMBI knockdown significantly reduced the mRNA expression and protein levels of cyclin D1, cyclin D3, and CDK2 in Y-MESO-27 and Y-MESO-14 cells (Fig. 3A, B, C and D). In addition, CDK4 mRNA expression and protein level were downregulated in Y-MESO-27 cells (Fig. 3C and D).

As knockdown of CTGF suppressed both BAMBI expression and MM cell proliferation, we speculated that CTGF knockdown would also downregulate the expression of cell cycle regulators. Indeed, mRNA and protein levels of cyclin D3 and CDK2 were significantly reduced in Y-MESO-14 and Y-MESO-27 cells, while CDK4 expression was reduced only in Y-MESO-27 cells (Fig. 3E–H). Thus, BAMBI and CTGF appear to drive MM cell proliferation by upregulating or sustaining expression of cell cycle regulators including cyclin D3, CDK2, CDK4, and cyclin D1.

### Subcellular distribution of BAMBI in MM cells

To provide further clues to BAMBI function in the regulation of MM cell growth, we examined its subcellular distribution in the aforementioned MM cell lines as well as in the mesothelial MeT-5A cell line. MeT-5A is a human mesothelial cell line isolated from pleural fluids of non-cancerous individuals and stably immortalized by simian virus 40 [26]. All MM cell line cells exhibited high levels of BAMBI, while its level in MeT-5A cells was less than that in MM cell lines (Fig. S4A), consistent with upregulation in MM. At the subcellular level, BAMBI was observed mainly in cytosol and to a lesser extent in membranes of MeT-5A and Y-MESO-27 cells defined by overlap with N-cadherin immunostaining (Fig. S4B).

### High BAMBI expression is associated with reduced overall survival of mesothelioma patients

**Fig. 4:**
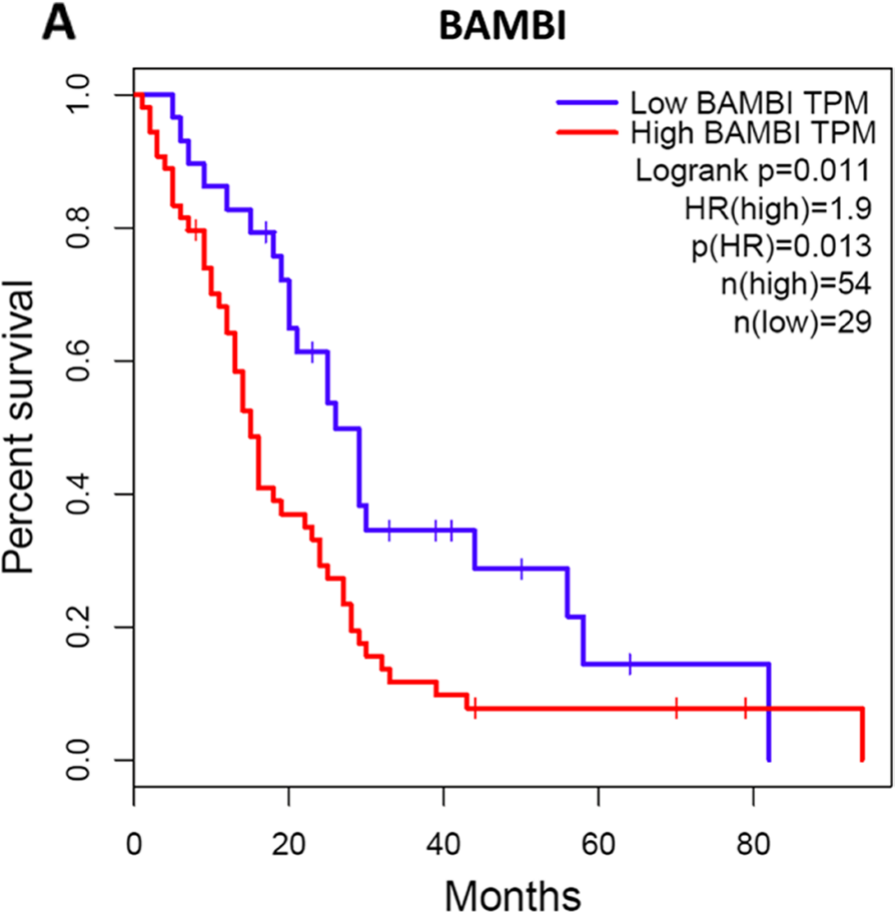
Elevated BAMBI expression predicts shorter overall survival of mesothelioma patients. **A**, Kaplan–Meier survival plots divided into tertiles were generated by software from the GEPIA webserver using mRNA sequence data from TCGA and GTEX. The samples were split into a high BAMBI expression group (*n* = 54) and a low BAMBI expression group (*n* = 29). Log-rank test indicate that overall survival was significantly reduced by high BAMBI expression (*p* < 0.05)

The reduction in MM cell proliferation by BAMBI knockdown suggests that BAMBI normally regulate cell proliferation, thereby increasing tumor aggression. To examine the influence of BAMBI expression on clinical outcome, we compared the Kaplan–Meyer survival curves of patients demonstrating high or low BAMBI mRNA expression. Consistent with promotion of tumor aggression, patients with high BAMBI expression had poorer prognosis than those with low BAMBI expression as reflect by overall survival (hazard ratio 1.9, 95% confidence interval, *p* = 0.011) (Fig. 4).

## Discussion

This study is the first study demonstrating the effect of CTGF/BAMBI axis on regulation of MM cell proliferation and thus adds to a growing body of literature identifying BAMBI as a potential treatment target for cancer therapy [31, 32]. Several cancer types appear to express elevated levels of BAMBI and these types are associate with poor prognosis [16, 29]. However, the physiological functions of BAMBI are still foggy. Although BAMBI is known to impede BMP and activin activity in Xenopus oocytes, genomic deletion did not generate developmental defects or reduce the postnatal survival of mice [15, 33]. Thus, the effects of BAMBI on BMP/TGF-β signaling and cell proliferation may be more important under pathological conditions than during development. Our results showed that expression of BAMBI was stronger in MM-derived cell lines than mesothelial-derived cells and its knockdown significantly suppressed MM cell proliferation (Fig. 2B and C, Fig. S4A). Notably that elevated BAMBI mRNA expression was associated with shorter survival of mesothelioma patients (Fig. 4).

BAMBI expression is regulated at the transcriptional and post-transcriptional levels by diverse signaling factors, including TGF-β, BMP, and β-catenin, and by specific pathophysiological conditions such hypoxia [28, 34–37]. Our results showed that BAMBI mRNA and protein expression levels were downregulated by CTGF knockdown (Fig. 1D), which reduced MM cell proliferation rate as same as the BAMBI knockdown (Fig. 1C). Recent studies have reported that RNAi-mediated silencing of CTGF or treatment with a specific antibody (pamrevlumab) in combination with pemetrexed, a single agent regimen in elderly mesothelioma patients, is effective for mesothelioma tumor suppression [11, 13]. These findings suggest that BAMBI participates in a signaling pathway with CTGF to regulate MM cell proliferation. It is thought that CTGF regulates cellular functions by modulating the activities of cytokines and growth factors such as TGF-β, BMP, and β-catenin [37, 38], which in turn may regulate transcription factors controlling BAMBI expression. We suggest that CTGF controls cancer cell growth at least in part by regulating BAMBI expression, although the detailed molecular pathways remain unclear. In Y-MESO-27 cells, BAMBI knockdown also increased CTGF expression (Fig. 2D), suggesting that BAMBI exerts negative feedback control of CTGF, possibly through modulation of BMP signaling. However, this effect was not observed in Y-MESO-14 cells, suggesting that CTGF expression is regulated by other more dominant factors in specific cell types and contexts [39].

Although BAMBI was first described as a transmembrane protein, many recent studies have also documented BAMBI expression in the cytosol and nucleus [40–42]. In mesothelioma and mesothelial cells as well, BAMBI immunofluorescence staining was observed mainly in the cytosol and to a lesser extent in the cell membrane (Fig. 2B), suggesting additional signaling functions aside from regulation of TGF-β/BMP signaling by acting as a pseudoreceptor. For example, BAMBI has been found to modulate TGF-β signaling in ovarian cancer cells by shuttling between the cytoplasm and nucleus together with Smads or regulate Wnt/ß-catenin signaling in human embryonic kidney (HEK)293 T cells [41, 43]. These findings were explained by limited homology between TGF-β superfamily receptor type I and BAMBI. However, BAMBI shares 6.1% and 9.1% homology with BMP receptor-IA and -IB, respectively [40, 43], while 10% is generally accepted as the minimum for prediction of shared function. Since TGF-β plays an important role in promoting cell proliferation and malignancy of MM cells through CTGF protein expression (11), we have checked the alteration of Smad2/3 phosphorylation and transactivation of TGF-βgenes in BAMBI knocked down Y-MESO-14 and Y-MESO-27 cells. Although we could not observe the obvious change in those pathways, we need to further examine the involvement of TGF-β/BMP signaling in regulation of cell proliferation by BAMBI.

Mesothelioma cell lines have a tumorigenic origin but still maintain cell cycle control mechanisms. Thus, these cells are suitable for investigation of novel therapies to alter the cell cycle of MM cells and suppress tumor growth [44, 45]. Among these strategies, targeting of cyclins and cyclin-dependent kinases has been examined, and selective CDK4/6 inhibitors have shown promising results [46]. Accumulated evidence also suggests that CTGF and BAMBI influence cell cycle progression by regulating the expression of multiple cell cycle proteins. For example, CTGF was reported to upregulate cyclin A in fibroblast [47], modulate the response of human mesangial cells to cyclin D1 and CDK inhibitors [48], and increase cyclin D3 expression in pancreatic beta cells [49]. BAMBI has also been reported to regulate cyclin D1, CDK2, and CDK6 in human osteosarcoma cells [30]. In the current study, knockdown of either CTGF or BAMBI reduced cyclin D3, CDK2, and CDK4 expression in MM cells (Fig. 3), and BAMBI knockdown also decreased the level of cyclin D1. The growth of MM cell is associated with enhanced CDK4 expression [50]. An antisense oligonucleotide targeting cyclin D1 was found to inhibit the proliferation of MM cells concomitant with suppression of cyclin D1, cyclin D3, and CDK2 synthesis [51, 52]. The similar effects of BAMBI and CTGF on cell cycle protein expression further support functions in MM cell proliferation. Our findings suggest that BAMBI promote MM cell proliferation and tumor aggression by acting downstream of CTGF. Moreover, BAMBI negatively regulate CTGF, possibly by interfering with TGF-β signaling and both BAMBI and CTGF may promote MM cell proliferation by activating or maintaining expression of multiple cell cycle regulators.

## Conclusions

In conclusion, here, we demonstrated that CTGF controls MM cell proliferation by regulating cell cycle progression through a signaling pathway involving BAMBI. We also present evidence that BAMBI acts not only as a downstream target of CTGF but as a negative feedback regulator. Finally, we report that elevated BAMBI expression is associated with poor clinical outcomes in mesothelioma patients, highlighting BAMBI as a potential molecular target for treatment.

## Supplementary Information

**Additional file 1:** **Figure S1.** Relative CTGF and BAMBI mRNA levels in MM cells knocked down by transfection of CTGF-targeted siRNA. Relative mRNA expression levels of BAMBI in MSTO-211H, NCI-H28, Y-MESO-8D, NCI-H2052 and NCI-H2452 cells transfected with a CTGF-targeted siRNA (20nM) are shown. BAMBI mRNA expression level was downregulated by transfection of CTGF-targeted siRNA in MSTO-211H, NCI-H28, Y-MESO-8D and NCI-H2052 cells, but not in NCI-H2452 cells. **Figure S2.** Relative BAMBI mRNA levels and cell proliferation rates of cells knocked down by Santa Cruz's BAMBI siRNA pool. A, Relative mRNA expression levels of BAMBI in Y-MESO-14 and Y-MESO-27 cells transfected with a Santa Cruz's BAMBI siRNA pool (20nM). BAMBI mRNA expression level was downregulated by transfection of siRNA in Y-MESO-14 and Y-MESO-27 cells. B, Graphs of proliferation rates as measured by viable cell counting assay. **Figure S3.** Knockdown of BAMBI does not affect the TGF-β/Smad activation in Y-MESO-27 cells. Western blotting showed p-Smad2 and p-Smad3 levels in Y-MESO-27 cells. Cells were transfected with a BAMBI-targeted siRNA (20 nM) and compared with nontransfected cells and cells transfected with control siRNA. Cells were stimulated by adding exogenous TGF-β1 and compared with cells without exogenous TGF-β1. **Figure S4.** Expression and subcellular localization of BAMBI protein in mesothelial and MM cell lines. A, Western blots showing BAMBI levels in seven MM cell lines and the mesothelial MeT-5A cell line. BAMBI protein was ubiquitously expressed by all mesothelioma cell lines but only weakly or below detection limits by MeT-5A cells. B, Immunofluorescence staining of Y-MESO-27 and MeT-5A cells showing that BAMBI localizes to the plasma membrane and cytosol. The nuclei were counterstained with DAPI (blue). The white arrows demarcate BAMBI expression in the cell membrane. Scale bars: 10 μm. **Table S1.** the oligonucleotide sequences of the siRNAs used in the study. **Table S2.** the oligonucleotide sequences and accession number of primers used in the study.

**Additional file 2:** **Table S3. **

**Additional file 3.** Unprocessed western blot images for Fig. 1. Unprocessed western blot images for Fig. 2. Unprocessed western blot images for Fig. 3. Unprocessed western blot images for Figure S3. Unprocessed western blot images for Figure S4.

## Data Availability

All data generated or analyzed during this study are included in this published article and its supplementary files.

## Abbreviations

MM: Malignant mesothelioma
CTGF: Connective tissue growth factor
BAMBI: Bone morphogenetic protein and activin membrane-bound inhibitor
CDK: Cyclin-dependent kinase
EMT: Epithelial-to-mesenchymal transition
TGF-β: Transforming growth factor beta
CRC: Colorectal cancer
SiRNA: Small interfering RNA
RT-qPCR: Real-time quantitative reverse transcription PCR
cDNA: First-strand complementary DNA
TCGA: Cancer Genome Atlas
GTEx: Genotype tissue expression
SD: Standard deviation
