# Supplementary material for: BMP and activin membrane-bound inhibitor regulate connective tissue growth factor controlling mesothelioma cell proliferation
Source: BMC Cancer. 2022 Sep 15;22:984. doi: 10.1186/s12885-022-10080-x (PMC9479400; doi:10.1186/s12885-022-10080-x)
Supplement: Supplementary file 1 — Additional file 1: Figure S1. Relative CTGF and BAMBI mRNA levels in MM cells knocked down by transfection of CTGF-targeted siRNA. Relative mRNA expression levels of BAMBI in MSTO-211H, NCI-H28, Y-MESO-8D, NCI-H2052 and NCI-H2452 cells transfected with a CTGF-targeted siRNA (20nM) are shown. BAMBI mRNA expression level was downregulated by transfection of CTGF-targeted siRNA in MSTO-211H, NCI-H28, Y-MESO-8D and NCI-H2052 cells, but not in NCI-H2452 cells. Figure S2. Relative BAMBI mRNA levels and cell proliferation rates of cells knocked down by Santa Cruz's BAMBI siRNA pool. A, Relative mRNA expression levels of BAMBI in Y-MESO-14 and Y-MESO-27 cells transfected with a Santa Cruz's BAMBI siRNA pool (20nM). BAMBI mRNA expression level was downregulated by transfection of siRNA in Y-MESO-14 and Y-MESO-27 cells. B, Graphs of proliferation rates as measured by viable cell counting assay. Figure S3. Knockdown of BAMBI does not affect the TGF-β/Smad activation in Y-MESO-27 cells. Western blotting showed p-Smad2 and p-Smad3 levels in Y-MESO-27 cells. Cells were transfected with a BAMBI-targeted siRNA (20 nM) and compared with nontransfected cells and cells transfected with control siRNA. Cells were stimulated by adding exogenous TGF-β1 and compared with cells without exogenous TGF-β1. Figure S4. Expression and subcellular localization of BAMBI protein in mesothelial and MM cell lines. A, Western blots showing BAMBI levels in seven MM cell lines and the mesothelial MeT-5A cell line. BAMBI protein was ubiquitously expressed by all mesothelioma cell lines but only weakly or below detection limits by MeT-5A cells. B, Immunofluorescence staining of Y-MESO-27 and MeT-5A cells showing that BAMBI localizes to the plasma membrane and cytosol. The nuclei were counterstained with DAPI (blue). The white arrows demarcate BAMBI expression in the cell membrane. Scale bars: 10 μm. Table S1. the oligonucleotide sequences of the siRNAs used in the study. Table S2. the oligonucleotide seque [file 12885_2022_10080_MOESM1_ESM.docx]

**Supplementary information**

**BMP and activin membrane-bound inhibitor regulate connective tissue growth factor controlling mesothelioma cell proliferation**

Nguyen Truong Duc Hoang^1*^, Ghmkin Hassan^1*^, Tomoya Suehiro^1^, Yuichi Mine^2^, Tohru Matsuki^3^, Makiko Fujii^1^

^1^Department of Genomic Oncology and Oral Medicine, Graduate School of Biomedical and Health Science, Hiroshima University, Hiroshima, Japan

^2^Department of Medical System Engineering, Division of Oral Health Sciences, Graduate School of Biomedical and Health Sciences, Hiroshima University, Hiroshima, Japan

^3^Department of Cellular Pathology, Institute for Developmental Research, Aichi Developmental Disability Center, Aichi Japan

^*^ Equal contribution.

**Correspondence to:** **Makiko Fujii**, Department of Genomic Oncology and Oral Medicine, Graduate School of Biomedical and Health Science, Hiroshima University, 1-2-3 Kasumi, Minami, Hiroshima, 834-8553, Japan


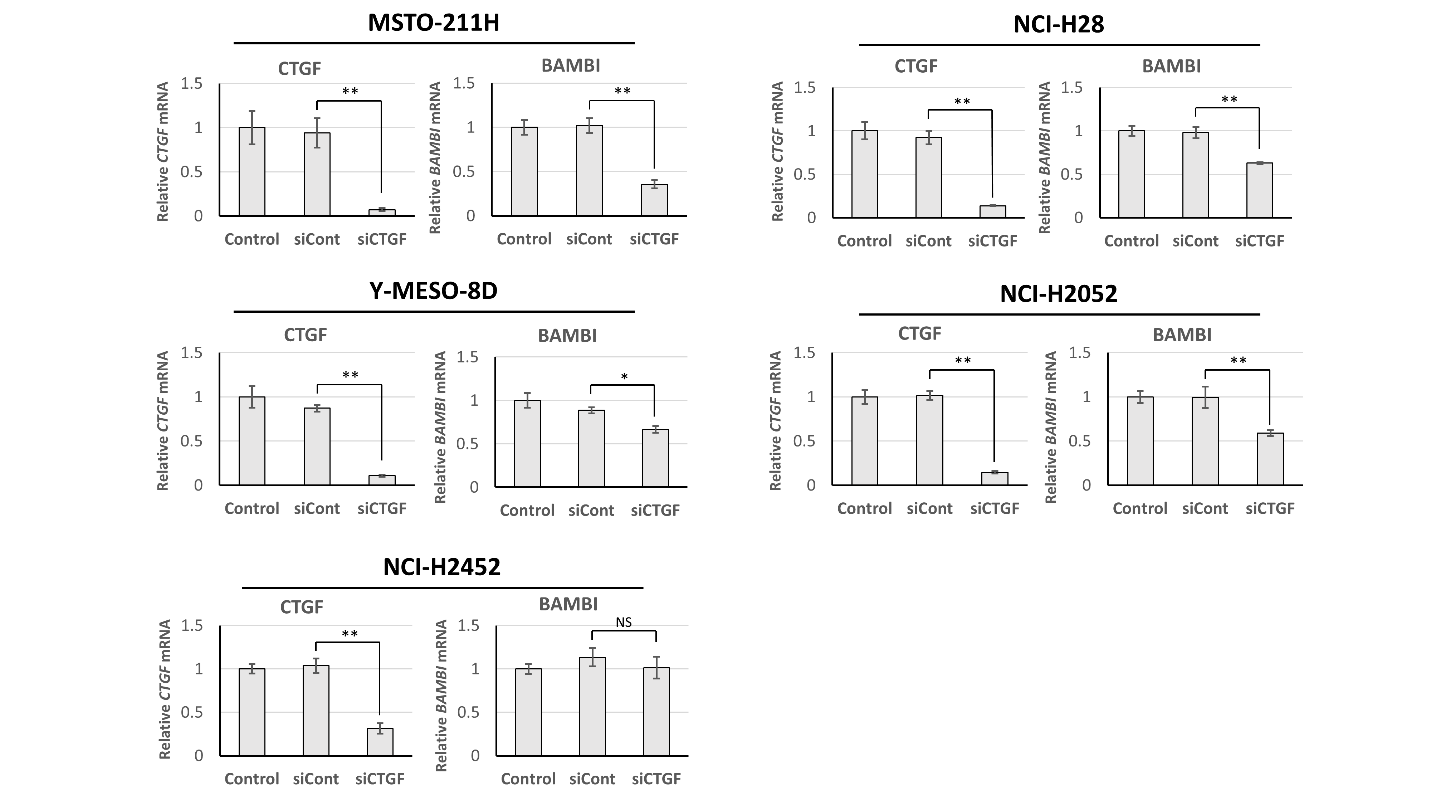


**Figure S1**, Relative CTGF and BAMBI mRNA levels in MM cells knocked down by transfection of CTGF-targeted siRNA.

Relative mRNA expression levels of BAMBI in MSTO-211H, NCI-H28, Y-MESO-8D, NCI-H2052 and NCI-H2452 cells transfected with a CTGF-targeted siRNA (20nM) are shown. BAMBI mRNA expression level was downregulated by transfection of CTGF-targeted siRNA in MSTO-211H, NCI-H28, Y-MESO-8D and NCI-H2052 cells, but not in NCI-H2452 cells.


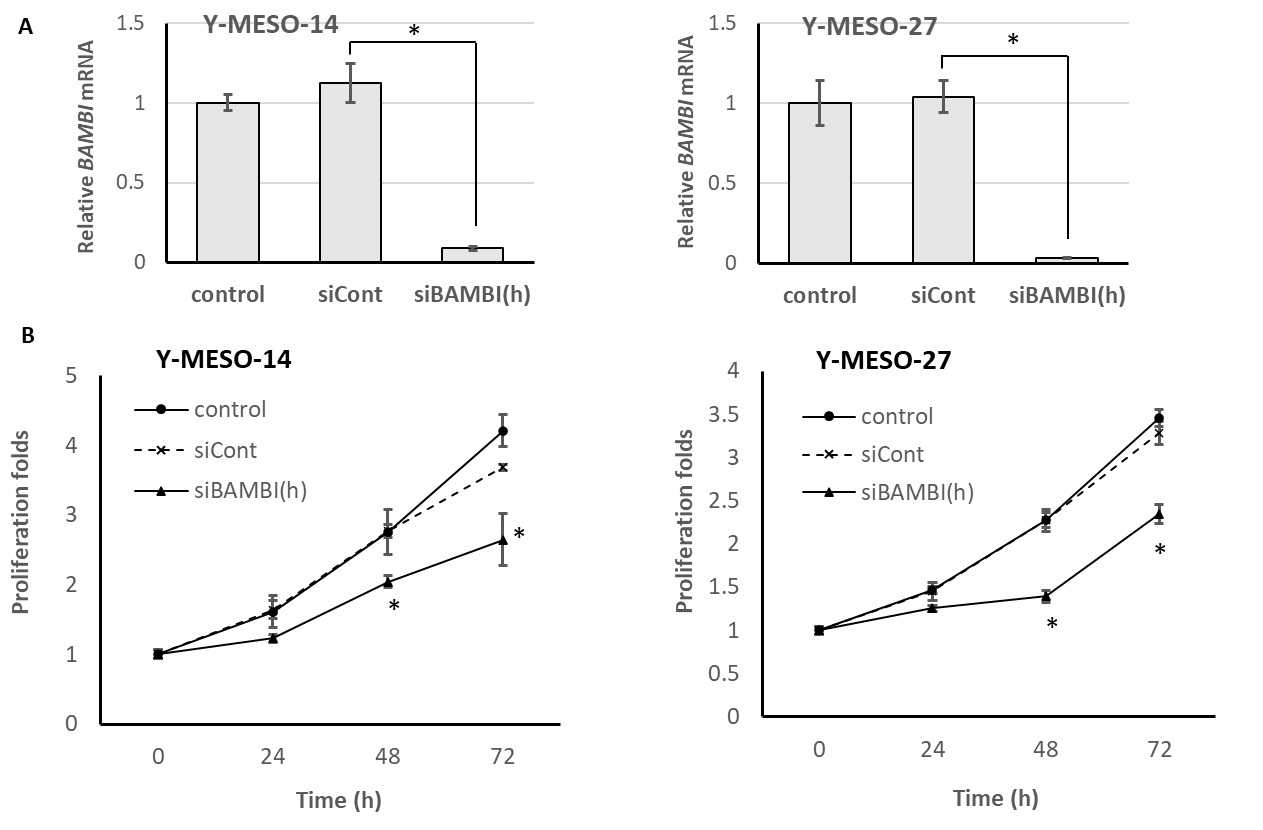


**Figure S2**, Relative BAMBI mRNA levels and cell proliferation rates of cells knocked down by Santa Cruz's BAMBI siRNA pool.

A, Relative mRNA expression levels of BAMBI in Y-MESO-14 and Y-MESO-27 cells transfected with a Santa Cruz's BAMBI siRNA pool (20nM). BAMBI mRNA expression level was downregulated by transfection of siRNA in Y-MESO-14 and Y-MESO-27 cells. B, Graphs of proliferation rates as measured by viable cell counting assay.


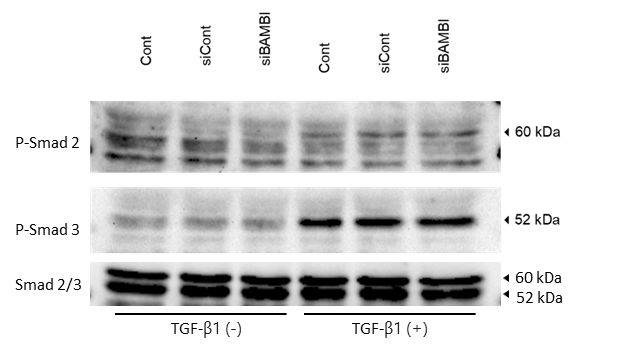


**Figure S3,** Knockdown of BAMBI does not affect the TGF-β/Smad activation in Y-MESO-27 cells.

Western blotting showed p-Smad2 and p-Smad3 levels in Y-MESO-27 cells. Cells were transfected with a BAMBI-targeted siRNA (20 nM) and compared with nontransfected cells and cells transfected with control siRNA. Cells were stimulated by adding exogenous TGF-β1 and compared with cells without exogenous TGF-β1.


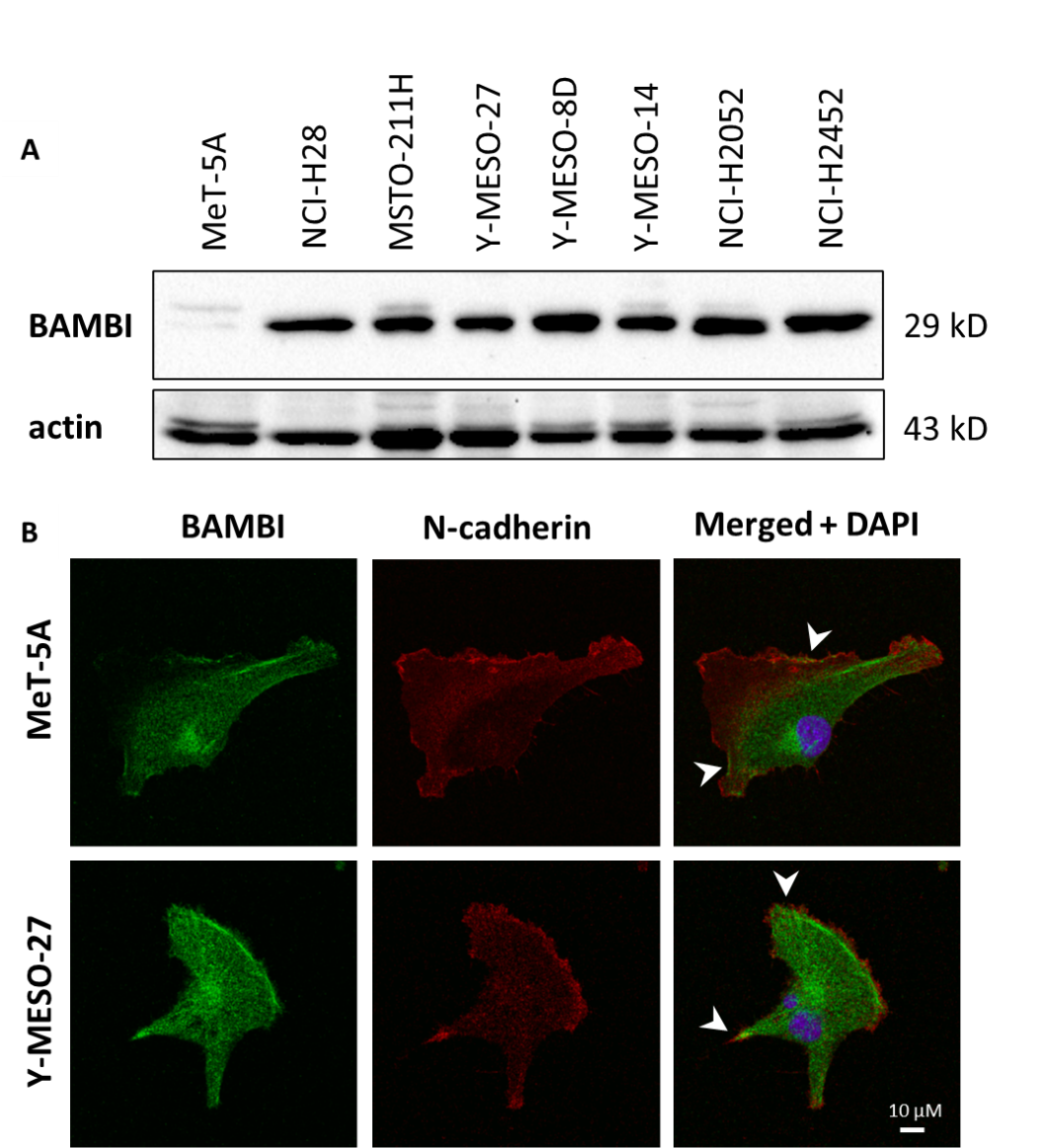


**Figure S4,** Expression and subcellular localization of BAMBI protein in mesothelial and MM cell lines.

A, Western blots showing BAMBI levels in seven MM cell lines and the mesothelial MeT-5A cell line. BAMBI protein was ubiquitously expressed by all mesothelioma cell lines but only weakly or below detection limits by MeT-5A cells. B, Immunofluorescence staining of Y-MESO-27 and MeT-5A cells showing that BAMBI localizes to the plasma membrane and cytosol. The nuclei were counterstained with DAPI (blue). The white arrows demarcate BAMBI expression in the cell membrane. Scale bars: 10 μm.

**Table S1,** the oligonucleotide sequences of the siRNAs used in the study.

| Smart pool | siRNA | Target Sequence |
| --- | --- | --- |
| CTGF  SO-2804612G | J-012633-10 | 5’-ACAAUGACAUCUUUGAAUC-3’ |
|  | J-012633-11 | 5’-AGGAAGAUGUACGGAGACA-3’ |
|  | J-012633-12 | 5’-CGAUUAGACUGGACAGCUU-3’ |
|  | J-012633-13 | 5’-GAGAGACAUUAACUCAUUA-3’ |
| BAMBI  SO-2889260G | J-019596-05 | 5’-AUAAGAGGCUGCAGGAUCA-3’ |
|  | J-019596-06 | 5’-UCACGGACACCAUUCCAAA-3’ |
|  | J-019596-07 | 5’-GAUCGCCACUCCAGCUACA-3’ |
|  | J-019596-08 | 5’-GGGGCAGGUUGCAAAGUUA-3’ |
| Control  SO-2855667G |  | 5’-UGGUUUACAUGUCGACUAA-3’ |
|  |  | 5’-UGGUUUACAUGUUGUGUGA-3’ |
|  |  | 5’-UGGUUUACAUGUUUUCUGA-3’ |
|  |  | 5’-UGGUUUACAUGUUUUCCUA-3’ |

**Table S2,** the oligonucleotide sequences and accession number of primers used in the study.

| Genes | Sequences for primers | Accession no. |
| --- | --- | --- |
| CTGF | 5’-CTGCGAGGAGTGGGTGTGT-3’  5’-GAACAGGCGCTCCACTCTGT-3’ | NM_001901.3 |
| BAMBI | 5’-CGATGTTCTCTCTCCTCCCAG-3’  5’-AATCAGCCCTCCAGCAATGG-3’ | NM_012342.3 |
| Cyclin D1 | 5’-GCTCCTGGTGAACAAGCTCAA-3’  5’-ATGGAGGGCGGATTGGAA-3’ | NM_053056.3 |
| Cyclin D3 | 5’-GGGACCTGGCTGCTGTGAT-3’  5’-GCGGGTACATGGCAAAGGTA-3’ | NM_001136017.3 |
| CDK2 | 5’-TGTGGTACCGAGCTCCTGAAA-3’  5’-AGATCCGGAAGAGCTGGTCAA-3’ | NM_001798.5 |
| CDK4 | 5’-CCGAGCTCCCGAAGTTCTTC-3’  5’-GCAGCCCAATCAGGTCAAAG-3’ | NM_000075.4 |
| GAPDH | 5’-CTCTGCCCCCTCTGCTGAT-3’  5’-CAGTCTTCTGGGTGGCAGTGA-3’ | NM_002046.7 |
