# Supplementary material for: BMP and activin membrane-bound inhibitor regulate connective tissue growth factor controlling mesothelioma cell proliferation
Source: BMC Cancer. 2022 Sep 15;22:984. doi: 10.1186/s12885-022-10080-x (PMC9479400; doi:10.1186/s12885-022-10080-x)
Supplement: Supplementary file 3 — Additional file 3. Unprocessed western blot images for Fig. 1. Unprocessed western blot images for Fig. 2. Unprocessed western blot images for Fig. 3. Unprocessed western blot images for Figure S3. Unprocessed western blot images for Figure S4. [file 12885_2022_10080_MOESM3_ESM.pdf]

Unprocessed western blot images for Figure 1

CTGF (Figure 1A)

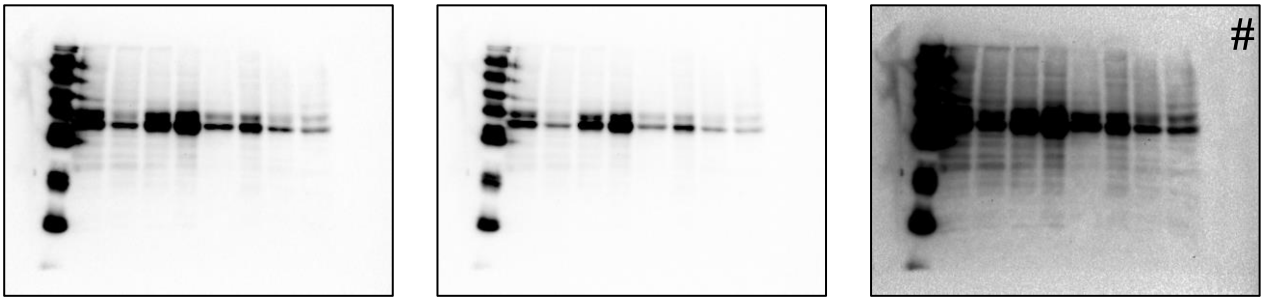

Different exposure

Actin (Figure 1A)

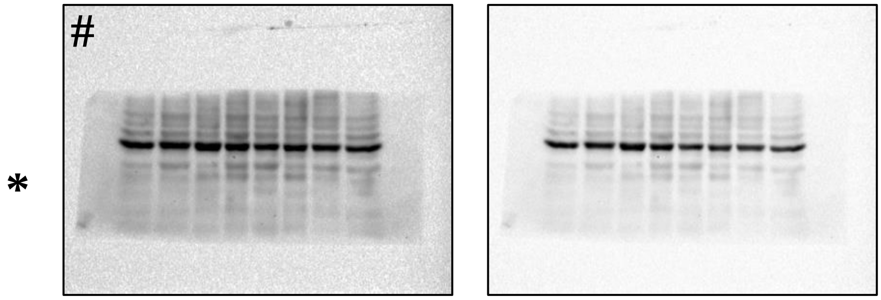

Different exposure

Con: control

siCon: siControl

SiCT: siCTGF

CTGF (Figure 1B)
Y-MESO-14

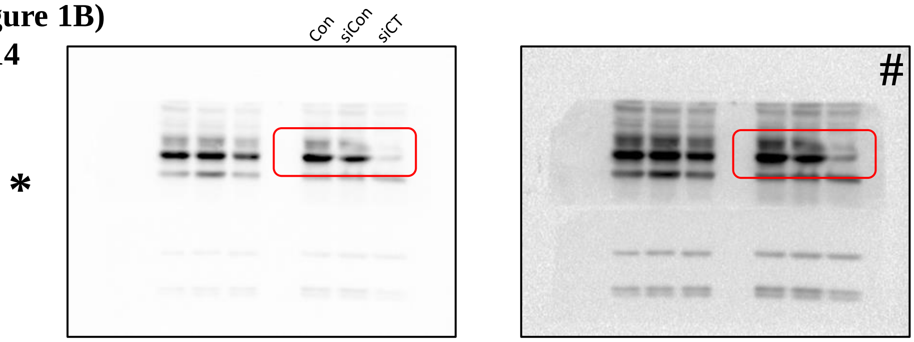

Different exposure

Actin (Figure 1B), Y-MESO-14

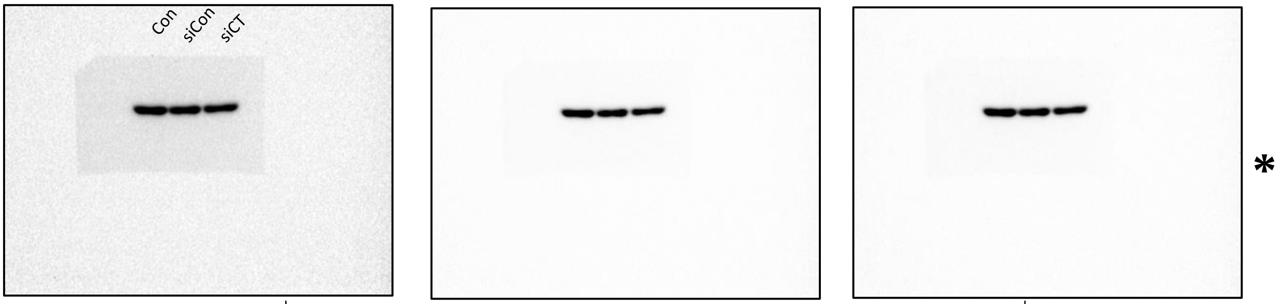

Different exposure

Actin and CTGF (Figure 1B), Y-MESO-27

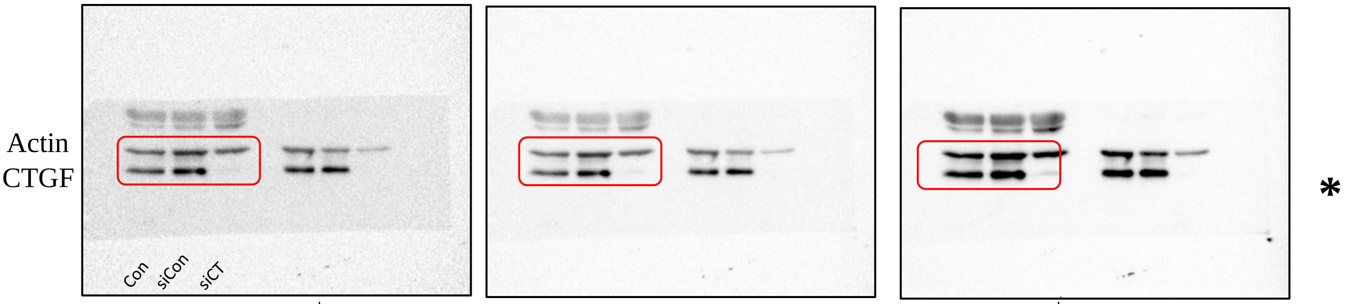

Different exposure

Background was adjusted in one blot to visualize membrane edges

* Blots were cut prior to hybridization with antibodies.

Unprocessed western blot images for Figure 1

BAMBI (Figure 1D), Y-MESO-27

*

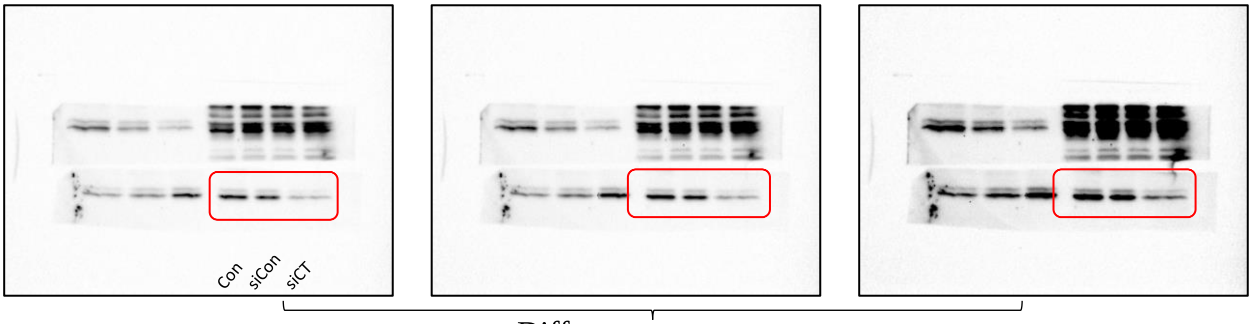

Different exposure

Actin (Figure 1D), Y-MESO-27

*

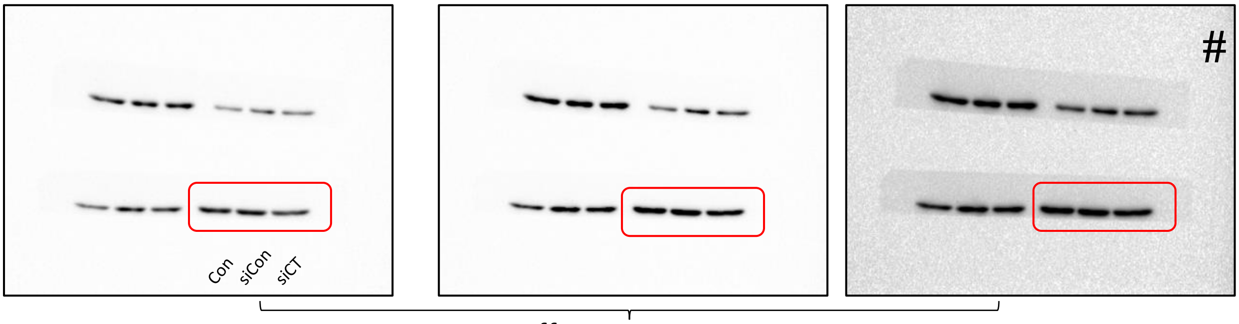

Different exposure

Background was adjusted in one blot to visualize membrane edges

* Blots were cut prior to hybridization with antibodies.

Unprocessed western blot images for Figure 2

CTGF (Figure 2 D)

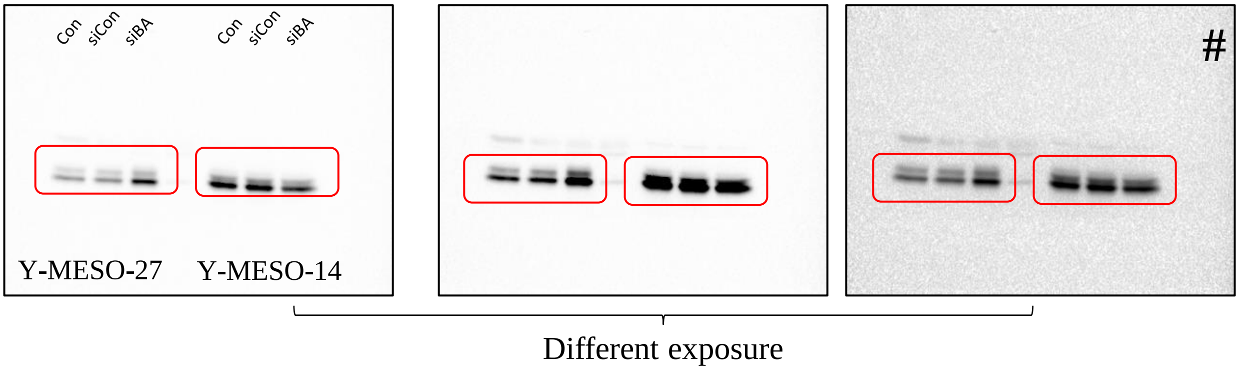

Actin (Figure 2 D)

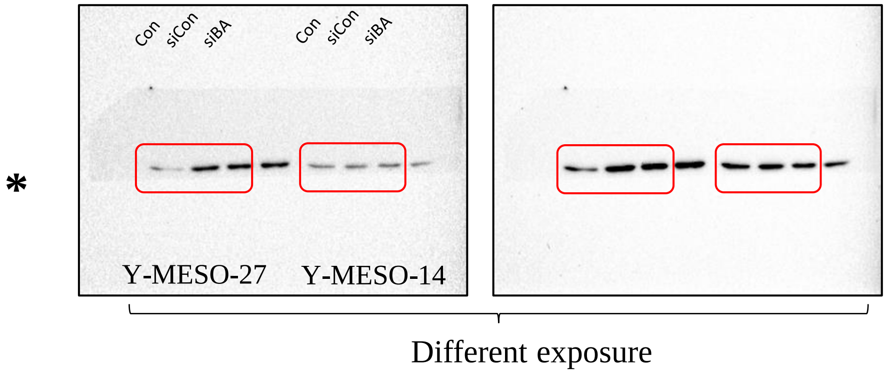

Con: control

siCon: siControl

SiCT: siCTGF

siBA: siBAMBI

Background was adjusted in one blot to visualize membrane edges

* Blots were cut prior to hybridization with antibodies.

Unprocessed western blot images for Figure 3

Y-MESO-14

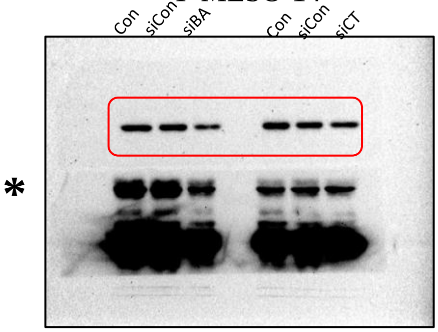

(Figure 3B, F) Cyclin D1

Y-MESO-14

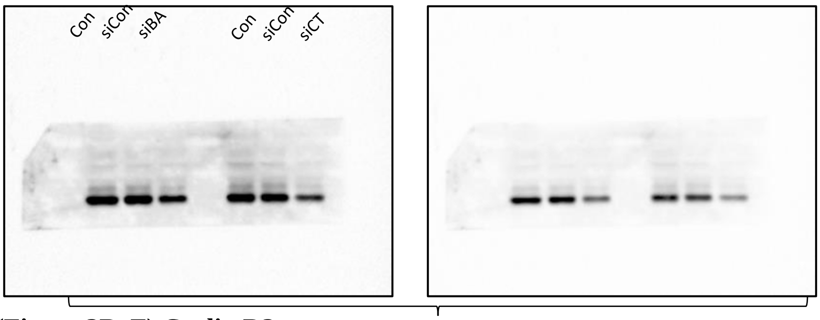

(Figure 3B, F) Cyclin D3   Different exposure

Y-MESO-14

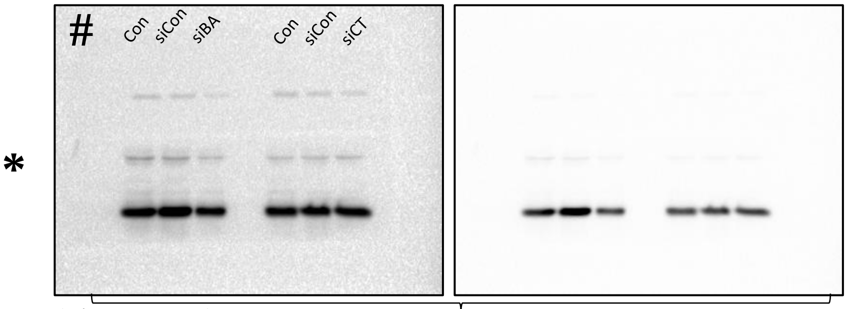

(Figure 3B, F) CDK4   Different exposure

Con: control
siCon: siControl
SiCT: siCTGF
siBA: siBAMBI

Y-MESO-14

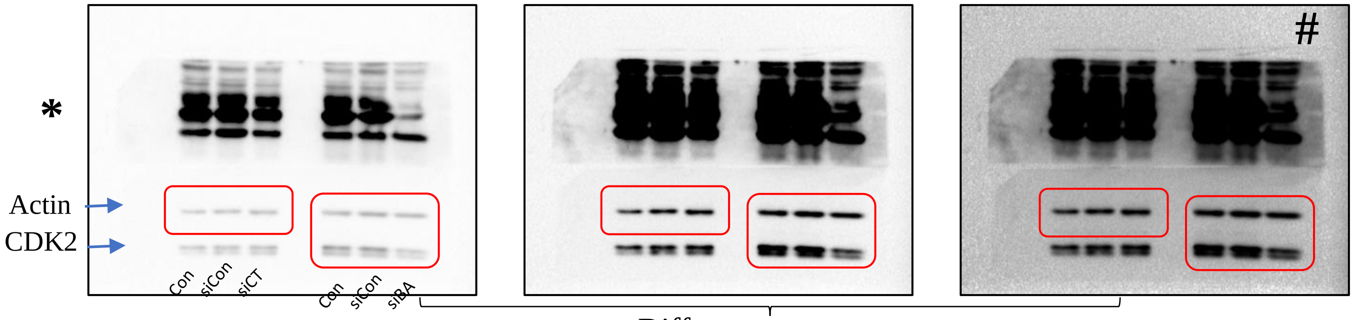

Actin (Figure 3B, F) and CDK4 (Figure 3B)   Different exposure

Y-MESO-14

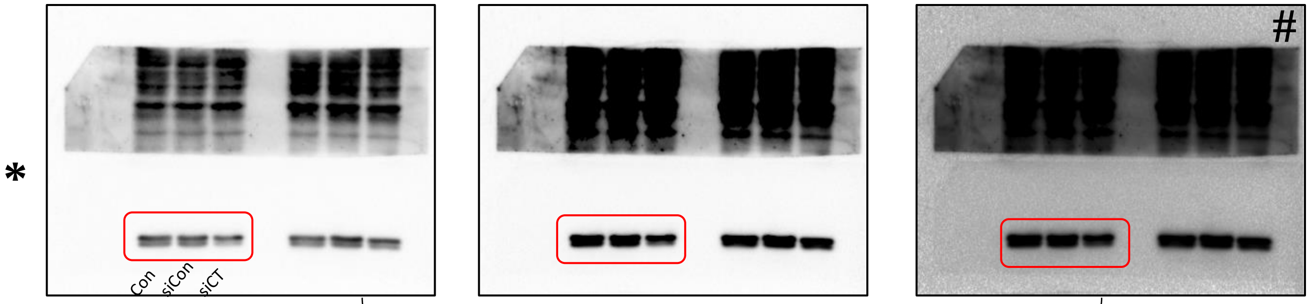

CDK2 (Figure 3F)   Different exposure

Y-MESO-27

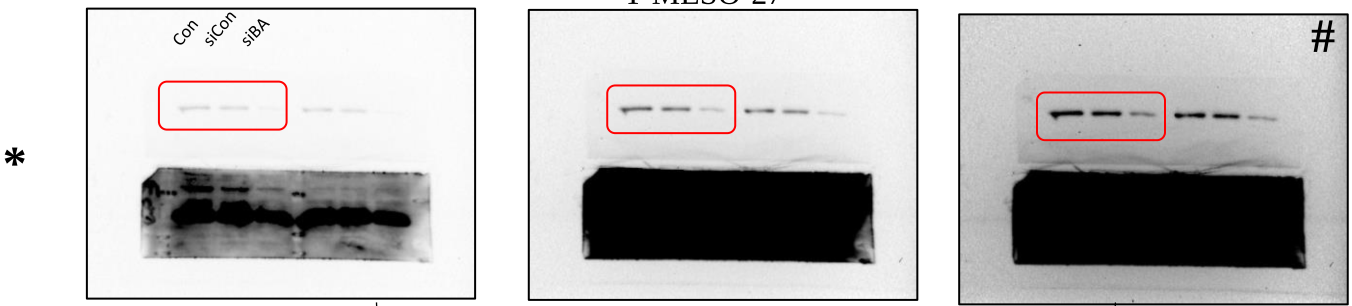

Cyclin D1 (Figure 3D)   Different exposure

Background was adjusted in one blot to visualize membrane edges   * Blots were cut prior to hybridization with antibodies.

Unprocessed western blot images for Figure 3

Y-MESO-27

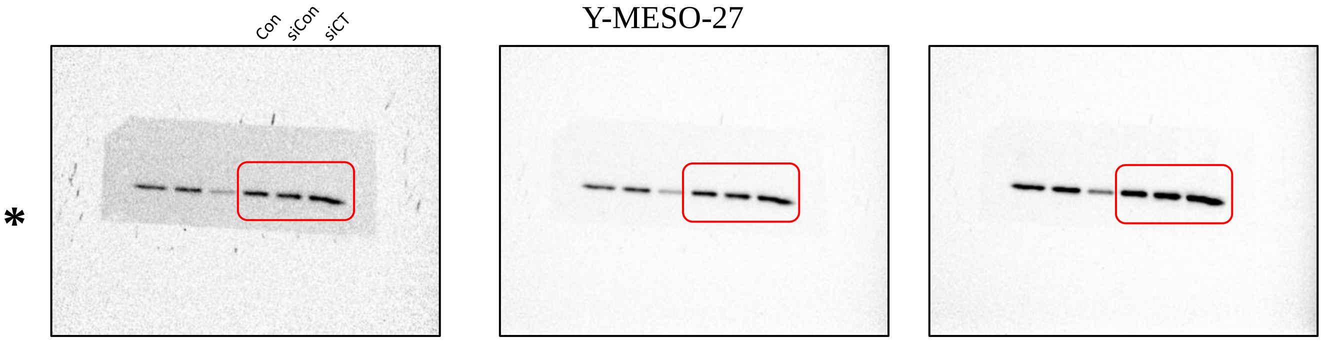

Cyclin D1 (Figure 3H)

Different exposure

Y-MESO-27

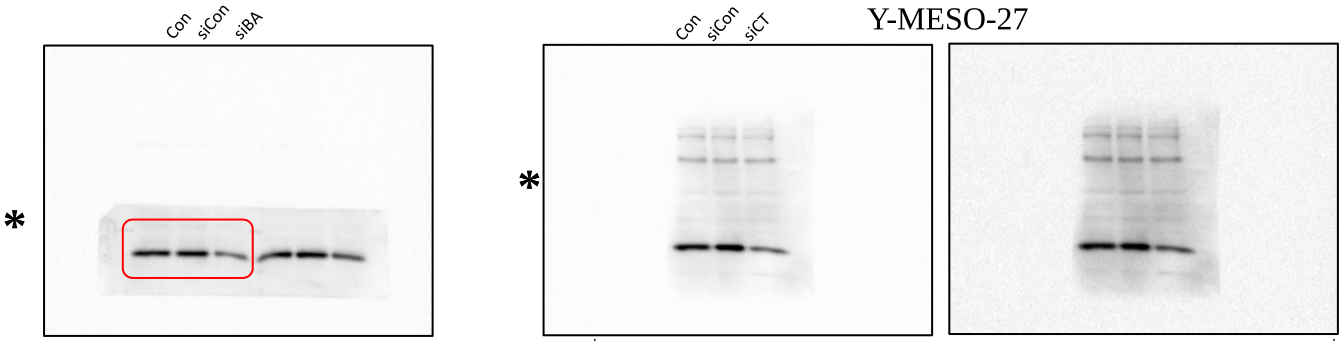

Cyclin D3 (Figure 3D)

Y-MESO-27

Cyclin D3 (Figure 3H)

Different exposure

Y-MESO-27

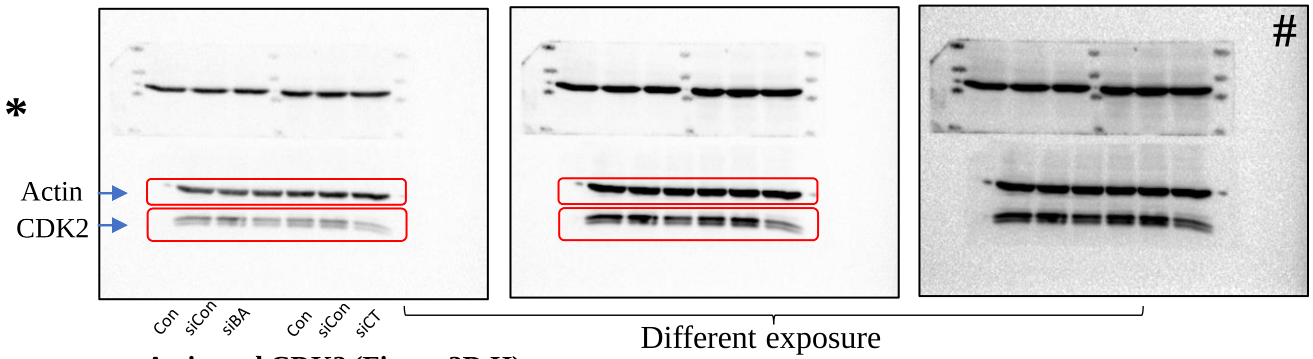

Different exposure

Actin and CDK2 (Figure 3D,H)

Y-MESO-27

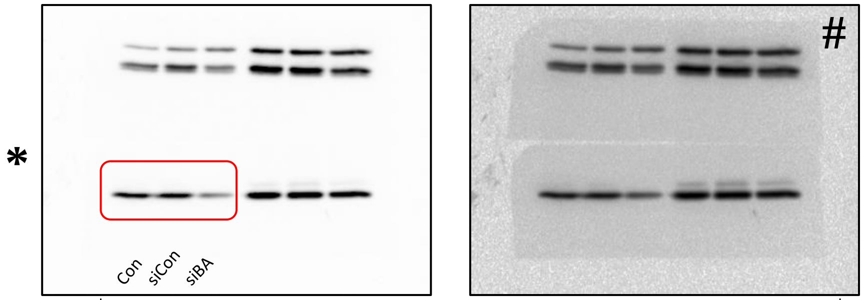

Con: control

siCon: siControl

SiCT: siCTGF

siBA: siBAMBI

CDK4 (Figure 3D)

Different exposure

merge

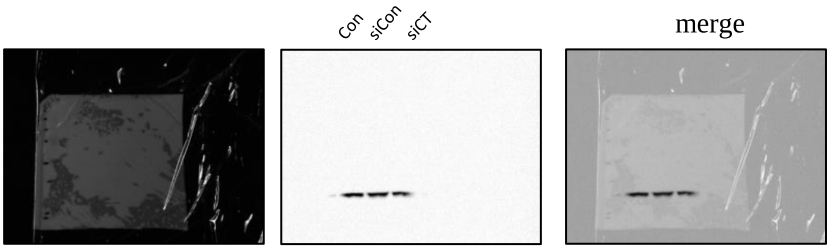

CDK4 (Figure 3H)

Y-MESO-27

Background was adjusted in one blot to visualize membrane edges

* Blots were cut prior to hybridization with antibodies.

Unprocessed western blot images for Figure S3

Smad 2/3 (Figure S3)

*

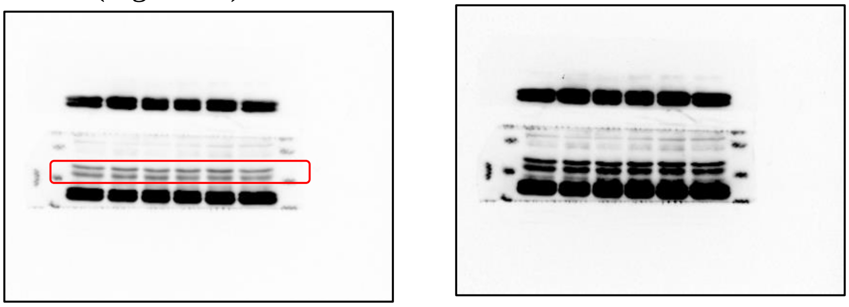

Different exposure

P-Smad 2 and P-Smad 3 (Figure S3)

*

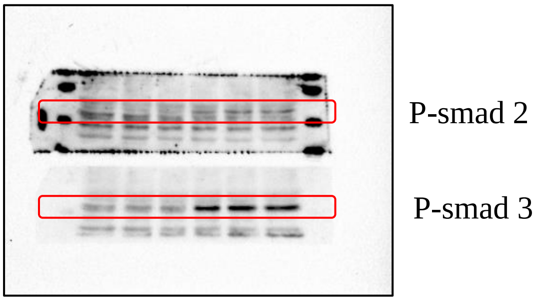

Unprocessed western blot images for Figure S4

BAMBI (Figure S4A)

*

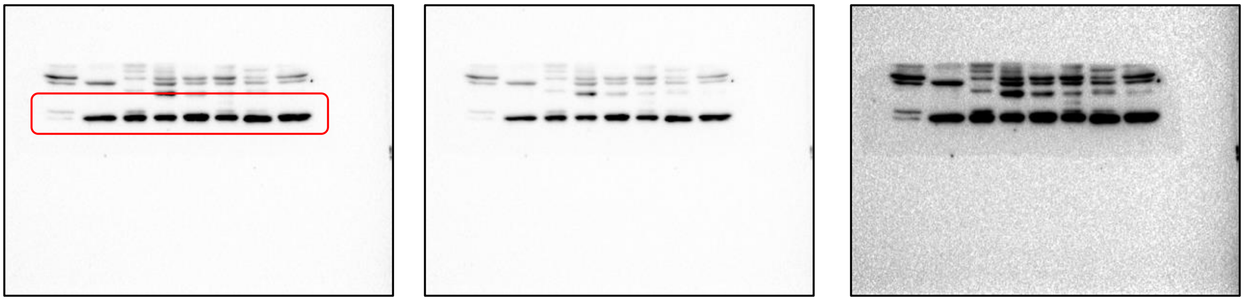

Different exposure

Actin (Figure S4A)

*

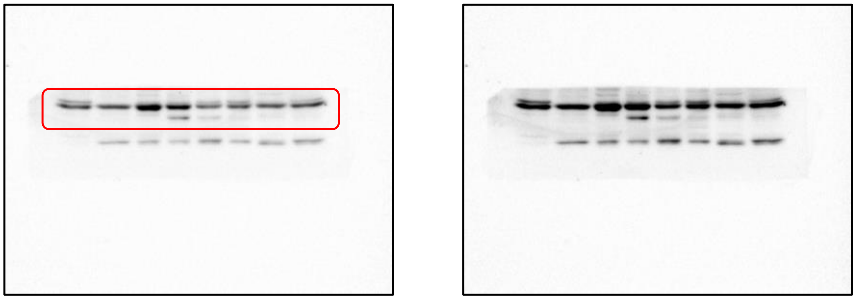

Different exposure

* Blots were cut prior to hybridization with antibodies.
